# Subchondral bone: An emerging target for the treatment of articular surface lesions of the knee

**DOI:** 10.1002/jeo2.12098

**Published:** 2024-07-21

**Authors:** Alessandro Sangiorgio, Luca Andriolo, Wayne Gersoff, Elizaveta Kon, Norimasa Nakamura, Stefan Nehrer, Francesca Vannini, Giuseppe Filardo

**Affiliations:** ^1^ Service of Orthopaedics and Traumatology, Department of Surgery EOC Lugano Switzerland; ^2^ Clinica Ortopedica e Traumatologica 2 IRCCS Istituto Ortopedico Rizzoli Bologna Italy; ^3^ Orthopedic Centers of Colorado Joint Preservation Institute, Clinical Instructor University of Colorado Health Sciences Center Aurora Colorado USA; ^4^ IRCCS Humanitas Research Hospital Rozzano Italy; ^5^ Department of Biomedical Sciences Humanitas University, Pieve Emanuele Milan Italy; ^6^ Department of Traumatology, Orthopaedics and Disaster Surgery Sechenov First Moscow State Medical University (Sechenov University) Moscow Russia; ^7^ Institute for Medical Science in Sports Osaka Health Science University Osaka Japan; ^8^ Center for Advanced Medical Engineering and Informatics Osaka University Suita Japan; ^9^ Faculty Health & Medicine University for Continuing Education Krems Austria; ^10^ Department of Orthopaedics and Traumatology University Hospital Krems, Karl Landsteiner University of Health Sciences Krems Austria; ^11^ Clinica Ortopedica e Traumatologica 1 IRCCS Istituto Ortopedico Rizzoli Bologna Italy; ^12^ Faculty of Biomedical Sciences Università della Svizzera Italiana Lugano Switzerland; ^13^ Applied and Translational Research (ATR) Center IRCCS Istituto Ortopedico Rizzoli Bologna Italy

**Keywords:** allograft, articular surface lesion, cartilage, subchondral bone, surgery

## Abstract

**Purpose:**

When dealing with the health status of the knee articular surface, the entire osteochondral unit has gained increasing attention, and in particular the subchondral bone, which plays a key role in the integrity of the osteochondral unit. The aim of this article was to discuss the current evidence on the role of the subchondral bone.

**Methods:**

Experts from different geographical regions were involved in performing a review on highly discussed topics about the subchondral bone, ranging from its etiopathogenetic role in joint degeneration processes to its prognostic role in chondral and osteochondral defects, up to treatment strategies to address both the subchondral bone and the articular surface.

**Discussion:**

Subchondral bone has a central role both from an aetiologic point of view and as a diagnostic tool, and its status was found to be relevant also as a prognostic factor in the follow‐up of chondral treatment. Finally, the recognition of its importance in the natural history of these lesions led to consider subchondral bone as a treatment target, with the development of osteochondral scaffolds and procedures to specifically address osteochondral lesions.

**Conclusion:**

Subchondral bone plays a central role in articular surface lesions from different points of view. Several aspects still need to be understood, but a growing interest in subchondral bone is to be expected in the upcoming future towards the optimization of joint preservation strategies.

**Level of Evidence:**

Level V, expert opinion.

**Abbreviations**
ACI: autologous chondrocyte implantation
AMIC: autologous matrix‐induced chondrogenesis
BMC: bone marrow aspirate concentrate
BMLs: bone marrow lesions
bTCP: beta‐tricalcium phosphate
HA: hydroxyapatite
iPSC: induced pluripotent stem cell
MACT: matrix‐associated autologous chondrocyte transplantation
MRI: magnetic resonance imaging
MSC: mesenchymal stem cell
OA: osteoarthritis
OCA: osteochondral allograft
OCD: osteochondritis dissecans
PRP: platelet‐rich plasma
STIR: suppression and short tau inversion recovery
TEC: tissue‐engineered construct

## INTRODUCTION

Historically, the cartilage layer has been the focus when dealing with the health status of the knee articular surface. From a physiological point of view, the peculiar anatomical structure and characteristics of articular cartilage were associated with the peculiar functions required by this joint [14], while from a pathological point of view, its limited healing potential [44, 45, 46] was considered directly responsible for the progression from a focal defect to extensive joint damage [93]. This clear and simple theory has been recently questioned. Joints are now considered as organs, not as a simple ensemble of tissues, a complex system composed of cartilage, bone, synovia, capsule, menisci, and ligaments. In this new perspective, the osteochondral unit gained increasing attention, and in particular the subchondral bone, which plays a key role in the integrity of the entire osteochondral unit. Its unique anatomical structure is well suited to support the articular cartilage mechanically while also providing nutrition for its basal layers [92].

The etiopathogenetic role of the subchondral bone has been extensively studied as well: in fact, it may be involved primarily in cases of osteochondritis dissecans (OCD) [53, 67], osteonecrosis, or trauma [73, 102]. Even focal chondral defects, if left untreated, may extend secondarily to the underlying subchondral bone, either with pathological changes such as overgrowth and sclerosis, or with bone loss [40, 41]. Consequently, treatment strategies shifted to a careful consideration of both cartilage and bone reconstruction, when approaching the treatment of chondral and osteochondral lesions [93]. Addressing the entire osteochondral unit is more challenging than treating the chondral layer alone, due to the different healing capacities of cartilage and bone. In recent years, the new achievements in tissue engineering led to the development of biomaterials that are able to target even such complex lesions, where previous treatments were not indicated. The aim of this article is to discuss the current evidence on the subchondral bone, ranging from its etiopathogenetic role in the joint degeneration processes to its prognostic role in chondral and osteochondral defects, up to the treatment strategies to address both the subchondral bone and the articular surface.

## SUBCHONDRAL BONE CHANGES IN JOINT DEGENERATIVE PROCESSES

**Figure 1:**
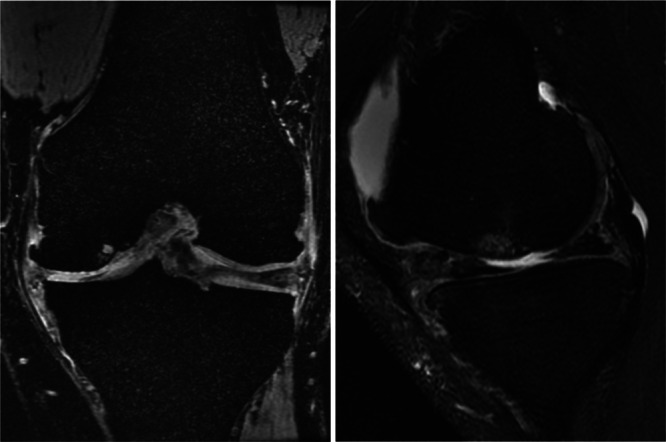
Subchondral lesions in osteoarthritic knee. In this example of magnetic resonance imaging (MRI) from a 51‐year‐old male patient with knee osteoarthritis (OA), the loss of articular cartilage is associated with subchondral damage, bone marrow oedema‐like lesions, and bone cysts.

Subchondral bone is known to play a crucial role in the initiation and progression of osteoarthritis (OA) [18]. Although subchondral bone has been defined in a number of ways, the term most often refers to the bony components lying distal to the calcified cartilage [16, 74]. It consists of an intricate structure composed of a dome‐like subchondral plate and underlying trabeculae, which is strictly related to the overlying cartilage from both a biomechanical and a biochemical point of view [71]. Strong evidence associates subchondral bone alterations with cartilage damage and loss in OA [71, 114]. From a histological point of view, despite the increase in bone volume fraction, subchondral bone in the course of OA is hypomineralized due to abnormal bone remodelling. Other histopathological changes, including microdamage, bone marrow oedema‐like lesions, and bone cysts, have been described [71] (Figure 1).

The role of subchondral bone in the development of OA is supported by the fact that the process of articular cartilage degeneration accompanying OA is preceded by subtle changes in the trabecular bone structure and the formation of new bone at the joint margins, called osteophytes [49, 50]. The diagnostic role of subchondral bone for OA is recognized also in the most used OA grading systems, such as the Kellgren‐Lawrence [51] or the OARSI systems [97], which include, besides descriptive denominators of joint space, deformity, formation of osteophytes as well as subchondral sclerosis. This represents the most problematic parameter because there is no clear definition of this term in the scientific literature. Some authors define subchondral sclerosis as a thickening of the subchondral bone plate, while others define it as a more visible structured architecture of the spongious bone [20].

Even without a consensus about the best way to characterize such alterations, changes to the trabecular bone structure have been demonstrated to appear in the subchondral bone compartment of the tibia even years before changes in articular cartilage thickness are detectable [13]. Consequently, subchondral bone is being investigated from a diagnostic point of view as a potential predictor of OA. In particular, a great effort has been expended in the analysis of the trabecular structure of the tibial head, with some studies investigating the fractal signature of the bone texture of the subchondral part of the tibia‐head obtained from radiographs [96]. The application of fractal analyses for the subchondral bone microarchitecture is based on the fact that trabecular bone texture detectable on plain radiographs has been correlated directly to the underlying three‐dimensional trabecular bone structure [6, 68]. These findings show the possibility of developing a low‐cost, radiographic‐based method for predicting the progression and incidence of OA by the use of computerized assessment of digital radiographic images [85]. This could help detect and study early articular changes, as well as guide the indication of the most suitable treatment to address the articular surface degeneration processes.

## SUBCHONDRAL BONE OEDEMA AND ARTICULAR CARTILAGE TREATMENT

Subchondral bone has been recently recognized to play an important role, even if not yet completely understood, in the decision‐making of articular surface treatments. The major interest of clinicians and scientists since the initial development of autologous chondrocyte implantation (ACI) was focused on developing different techniques for articular cartilage restoration. Similarly, the decision‐making process has always been centred on the status of the articular surface. Only recently the importance of the integrity of subchondral bone has been receiving increasing attention. The relationship between the subchondral bone and the articular cartilage is symbiotic in nature: the health of one depends upon the health of the other.

One of the most frequently observed alterations of the subchondral bone is represented by the subchondral bone marrow lesions (BMLs), also known as subchondral bone oedema [76], defined as an alteration of the MRI signal intensity of the bone marrow, seen on T1‐weighted and T2‐weighted images and best seen with fat suppression and short tau inversion recovery (STIR) sequences [66]. The presence of subchondral BML generally indicates some form of injury, which could be the result of acute trauma, metabolic abnormalities, transient overload secondary to articular cartilage injury, or chronic overload, as in OA. These injuries result in the disequilibrium of the stimulus and the bone's ability to remodel and restore the physiologic and functional condition. On MRI, this will be represented by alterations in the bone signal intensity. The pattern of this alteration may also differ depending upon the aetiology of the subchondral BML [66], and so will the prognosis and the possible treatment. The subchondral BML seen secondary to articular cartilage defects generally indicates a compromise in the health of the subchondral bone [7] and can represent either a reversible or irreversible process. An example of a reversible condition is represented by the typical bone bruise detected after anterior cruciate ligament rupture, whereas the BML seen in OA knees may lead to an insufficiency fracture or even to osteonecrosis, which represents an irreversible process [66, 75].

The challenging dilemma that must be addressed is whether or not an altered subchondral bone may support the healing of articular cartilage. Also, it must then be considered whether restoring the articular cartilage surface alone will provide enough protection for the altered subchondral bone to heal. Several chondral treatment procedures have been described over the years, each one of them presenting its own peculiar interaction with the presence of BML. One of the most used cartilage procedures is represented by microfractures, which stimulate cartilage healing by accessing the regenerative elements of bone marrow and penetrating the subchondral bone directly from within the defect. Its widespread use is due to the minimally invasive approach, limited surgical morbidity, and satisfying short‐ to mid‐term clinical results [1, 69, 107]. Nevertheless, if from one side the access to the subchondral bone provides regenerative elements allowing a surface repair with fibro‐cartilagineous tissue, on the other side, this sort of iatrogenic “damage” of the subchondral bone has also been correlated with the suffering of this structure over time. Both preclinical and clinical studies highlighted persistent alterations in the subchondral bone following microfractures, in particular in the deeper layer [100] with the reactive development of intralesional osteophytes [25], and the lasting morphological changes in the subchondral bone were even suggested to be indicative of long‐term failure of the microfracture procedure [34, 106]. Besides the direct injury to the subchondral bone inherent to the surgical procedure, a possible explanation of the subchondral alterations after microfracture may be found in the fibrous repair tissue provided, which does not match the mechanical properties of the cartilage tissue and may determine an altered load to the underlying bone [17]. Accordingly, even though no specific evidence is yet available, the indication of microfracture should be carefully considered in the presence of osteochondral lesions and cartilage lesions with subchondral bone oedema beneath. The association of microfracture to the use of a chondral cell‐free scaffold, like in the autologous matrix‐induced chondrogenesis (AMIC) technique [5], may overcome these limits by providing more stable support for cell growth, although the direct damage to the subchondral plate may still be significant, and the evidence in this sense is still lacking.

The damage to the subchondral plate caused by microfractures was shown to affect also the results of the chondral regenerative procedures like ACI, which do not imply direct damage to the subchondral bone. In fact, Minas et al. [82] analysed 522 chondral defects in 321 patients treated with ACI and demonstrated that patients who had previously undergone treatments affecting the subchondral bone had a failure rate three times higher than patients undergoing primary ACI. On cartilage lesions not previously treated, the more hyaline‐like tissue provided by ACI may better protect the subchondral bone from a mechanical point of view. Nevertheless, a persistency of subchondral bone alterations has been reported also for ACI and matrix‐associated autologous chondrocyte transplantation (MACT) [30]. However, the evidence on this matter is inconsistent when looking more in general at regenerative treatments. While in the shorter term, the presence of subchondral bone marrow oedema was a negative prognostic indicator for ACI, no influence on the failure rate or the clinical results at longer follow‐up has been demonstrated for MACT. In patients undergoing osteochondral allograft transplantation, more severe BML were correlated with higher post‐operative outcomes, but they may also be associated with an increase in graft failure [43]. The role of subchondral bone alterations in the treatment and healing process with other procedures developed through the years will be discussed more in detail in the following paragraphs.

## SUBCHONDRAL BONE LESIONS AFTER CARTILAGE SURGERY

**Figure 2:**
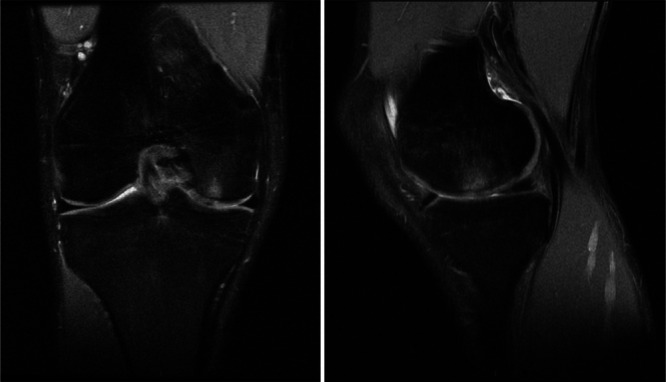
Subchondral lesions are a common finding after cartilage procedure. In this example, magnetic resonance imaging (MRI) of perifocal bone marrow oedema‐like signals are detected 5 years after the matrix‐associated autologous chondrocyte transplantation (MACT) procedure for the chondral lesion of medial femoral condyle in a 20‐year‐old male patient, but the patient presented a full symptomatic and functional recovery at the time of MRI.

Subchondral bone has been recently recognized for its role also in the altered joint environment after surgical treatment for chondral lesions. The definition of the changes at the subchondral bone level is important to understand the maturation and evolution of the treated articular compartments, but most of the studies on cartilage treatments actually lack data on subchondral bone changes to document their extent, frequency, timing of presentation and correlation with other factors, to understand their significance and their possible influence on the outcome of cartilage surgery. Perifocal bone marrow oedema‐like signals are frequent findings after both chondral and osteochondral surgical procedures, ranging from about 40% to 80% [87, 111] (Figure 2).

BML is detectable around and above the treated site, usually together with the signs of the surgical procedure itself, although without presenting specifically distinctive aspects [99]. From a histological point of view, subchondral bone cysts on one side and upward subchondral bone plate migration or intralesional osteophytes formation on the other side are subchondral bone alterations found after cartilage repair surgery [90]. Deteriorations of the subchondral microarchitecture, such as changes in bone mineral density, bone volume, and trabecular thickness, can also be found, suggesting that the entire osteochondral unit can be altered either as short‐term maturation result or as long‐term tissue evolution [91].

The etiopathology of these findings is still unclear, as well as their evolution, with both evidence of reduction or increase of its incidence over time. In a study aiming at clarifying the evolution of BML detected after cartilage surgery [30], BMLs were present in the first postoperative phases, markedly reduced at 2 and 3 years, and then again increased at mid/long‐term follow‐up. This peculiar trend could be the expression of the tissue modifications over time: the initial reduction could be explained by the maturation phase, which for such cartilage treatments is commonly acknowledged to stabilize at around 2 years. On the other hand, the tissue obtained as a result of cartilage procedures may not be sufficient to protect the subchondral bone from the joint mechanical forces, leading to progressive abnormal subchondral bone stimulation and suffering.

Despite the aforementioned findings shedding some light on both frequency and evolution, no correlation has been found between BML and clinical outcome, thus making the clinical significance questionable and of difficult interpretation [30, 87, 111]. The high MRI sensitivity might allow early changes to be detected, which may be a limited tissue reaction, that is abnormal but still not severe enough to affect the clinical outcome, even at mid/long‐term follow‐up. Nonetheless, BML is a common finding after cartilage surgery, and there is a need for a better understanding of the evolution of post‐surgical BML over time, as well as its importance as a prognostic factor.

More recently, the osteochondral unit has also become the focus of treatment procedures, which will be more extensively described in the next chapters. Several techniques have been proposed to treat simultaneously both the chondral and the subchondral bone layers, thus addressing at the same time both the articular cartilage defect and the underlying BML. Both osteochondral graft implantation (autograft or allograft) and osteochondral scaffolds demonstrated good results in treating osteochondral issues. However, these techniques are limited in terms of the depth of subchondral BML that can be addressed. The overall significance of the remaining BML is uncertain; it may resolve over time, and there is no evidence that even persistent subchondral bone alteration may compromise the results of an osteochondral graft.

Thus, the answer to the question about the relationship between BML and articular cartilage treatment is multifactorial and should involve the localization, aetiology, and chronicity of both the subchondral bone oedema and the articular cartilage defect. Algorithms should be developed to create a pathway for treating articular cartilage injuries in the presence of subchondral BML, as well as to interpret and address it when appearing post‐operatively. Important considerations during treatment may include the following: is the BML reversible or not; other potential sources of overloading (meniscal deficiency, malalignment, and ligamentous instability) need to be addressed; has the knee environment progressed to that of OA; what is the depth and width of the oedema; and is the subchondral oedema acute or chronic. The evaluation of these factors should lead to the choice of the most appropriate treatment considering the status of the subchondral bone. It is crucial to keep in mind that for some types of chondral and osteochondral lesions, osteochondral procedures are available to address the entire osteochondral unit.

## SUBCHONDRAL BONE AND OSTEOCHONDRAL ALLOGRAFTS

Osteochondral allograft (OCA) transplantation represents an attractive technique that allows for the simultaneous resurfacing of large articular defects and the correction of underlying bone abnormalities and represents a valid solution for symptomatic full‐thickness cartilage lesions greater than 2 cm^2^, large lesions with anatomy disruption, deep lesions with subchondral damage, and revision techniques when a previous surgical procedure has failed [72]. Besides the fact that cartilage is a relatively immune‐privileged tissue, it is widely known that bone has the potential to heal and remodel [19]. Previous reports supported the safety, efficacy, and survivorship of focal unipolar, multisurface unipolar, and bipolar OCA transplantations in the knee, with functional graft survival rates of up to 82% at 10 years, 74% at 15 years, and 66% at 20 years [37, 70, 108, 116]. Nevertheless, the larger part of the studies focused on the importance of chondrocyte viability and survivorship, while there has been relatively less interest in the osseous integration of OCA following transplantation [23, 80, 86]. Still, insufficient allograft bone incorporation is one of the two primary modes of failures after OCA, along with loss of transplanted cartilage integrity [110].

Subchondral bone integration is actually a key part of OCA success and is fundamental that the integration process, known as creeping substitution, occurs as smoothly and fast as possible [3, 8, 83]. Creeping substitution is a time‐demanding procedure, capable of occurring only in allografts of a limited thickness (less than 2 cm) and that takes several months. Especially in large grafts, a long time is needed in order to complete the process and make sure the graft is no more at risk of failure through graft breaking or mobilization [35, 36]. Up to now, no scoring system for cartilage repair techniques has taken into proper account the large impact that the transplanted subchondral bone has on the final result of osteochondral repair. Recently, Chang et al. developed a comprehensive osteochondral allograft MRI scoring system capable of integrating parameters already included in the widely used MOCART with some unique features of the transplanted subchondral bone. The new OCAMRISS score, in fact, takes into account parameters such as the congruity of the graft and host‐graft junction at the subchondral bone plate, subchondral bone marrow signal intensity of the graft relative to the epiphyseal bone, osseous integration at the host‐graft junction, and the presence of cystic changes of the graft and host‐graft junction. Furthermore, the system was validated with histopathologic, micro‐computed tomography, and biomechanical parameters and may be a valuable tool to evaluate the subchondral bone conditions following allografting [19].

Since a lack of OCA bone integration can be a major cause of treatment failure, methods for speeding up and enhancing OCA bone integration to mitigate this risk are highly desirable. Unfortunately, although immune responses associated with antigen mismatch may influence OCA bone healing and incorporation as evidenced by MRI, histologic, and micro toxicity analyses, there are still no indications about a cost‐effective therapy (in terms of morbidity), capable to reduce the immunoreaction of the implanted allograft and improve the integration process [88]. Giannini et al. applied a slight immunosuppressive protocol to ankle bipolar shell allografts, in order to improve osteointegration and cartilage viability overtime, without appreciable effects [38]. Currently, the optimal standard‐of‐care methods for mitigating these effects include the use of shell grafts with minimal‐thickness subchondral bone and pulsed lavage of fresh OCAs to reduce allograft immunogenicity by diluting allogeneic bone marrow elements in the graft [3].

Bone marrow aspirate concentrate (BMC), which contains osteoprogenitor cells and osteoinductive proteins, is approved for clinical use in augmenting bone healing. BMC potentiates an anabolic and anti‐inflammatory environment that may accelerate and promote the process of OCA bone integration [22]. Based on this knowledge, Stoker et al. compared in vitro the potential of BMC and leuko‐reduced platelet‐rich plasma (PRP) to repopulate the osseous portion of an OCA with cells and deliver osteogenic proteins. The BMC used in the study contained osteoprogenitor cells that were capable of repopulating the osseous portion of OCAs. Viable cell repopulation of OCAs did not occur for any PRP‐ or saline‐treated grafts, indicating that only BMC provided the potential for enhancing OCA bone integration by seeding OCAs with autologous osteoprogenitor cells [109]. Clinical data regarding the improvement of OCA integration by the use of BMC in the knee are still very limited. In 2017, Lasun et al. [89] published a series of patients enrolled in a prospective registry who were treated with transplantation of large OCAs to one or both femoral condyles. Patients were stratified into two groups based on BMC treatment versus no BMC treatment; the treatment was non‐randomized. The study described 17 condyles in 15 patients who underwent OCA transplantation without BMC and 29 condyles in 22 patients who underwent OCA transplantation with BMC. The BMC group had significantly higher graft integration scores at 6 weeks, 3 months, and 6 months after surgery. Graft sclerosis was significantly less in the BMC group at 6 weeks and 3 months, with no significant difference at 6 months after surgery. Nevertheless, the role of BMC in improving OCA bone healing is still controversial; although small case series and several limitations in the study, Wang et al. recently examined retrospectively patients treated with OCA + BMC or OCA alone for full‐thickness chondral defects of the distal femur both clinically and by MRI and reported no significant differences in the demographics or lesion characteristics between treatment groups in either postoperative phase [115]. Future studies are needed to better understand how to improve OCA integration.

## IMPORTANCE OF SUBCHONDRAL BONE INTEGRITY FOR OSTEOCHONDRAL RESTORATION BY TISSUE‐ENGINEERED APPROACHES

The limits of allografts, especially in terms of availability, pushed the research in the direction of osteochondral tissue‐engineered approaches. To understand the reasons that led to defining the characteristics of osteochondral tissue‐engineering approaches, a step back is needed towards the biological and mechanical properties of the osteochondral complex. As already defined, it consists of both the articular cartilage and the underlying subchondral bone, with biochemical and biomechanical differences characterizing these two structures [104]. Biochemically, the cartilage tissue largely comprises water, chondrocytes, type II collagen, and proteoglycans, while the bone is a complex tissue consisting mainly of collagen type I and hydroxyapatite (HA), with these components providing the bony stiffness and compressive strength. The compressive modulus of the subchondral bone is higher than that of cartilage. Therefore, it is reasonable for an ideal repair of osteochondral lesions to facilitate zonal restoration of cartilage and subchondral bone, layer by layer, mimicking the natural articular structure [47, 103]. Accordingly, to regenerate these structures in a layer‐by‐layer fashion, biphasic or triphasic constructs have been developed, and such constructs have been reported to be feasible for osteochondral repair in vivo [55, 78, 103].

The successful regeneration of the subchondral bone is a key point for an effective osteochondral repair [39, 82, 105]. Therefore, for an effective subchondral bone repair, it is critically important to choose suitable materials with initial mechanical strength and good potential for bone ingrowth and integration of native surrounding bone, which are characteristics that will promote subchondral bone repair accompanied by good tissue quality, both morphologically and biomechanically. Several materials, including ceramics, natural materials, and synthetic polymers, have been tested for scaffolds of the subchondral bone layer [104]. On the other hand, the optimization and selection of biomaterials for subchondral bone repair have not been fully investigated. Thus, the ideal structure and composition of bioimplants that repair osteochondral lesions have not been fully elucidated. Further studies will be needed and should be conducted in a methodologically rigorous fashion.

Recently, various ceramic bone substitutes with improved biocompatibility have been developed. Among them, HA and beta‐tricalcium phosphate (bTCP) have been widely used in clinical practice. In a previous study [118], it was found that the compressive strength of the repair bone after implantation with an HA ceramic gradually increased and remained at the highest level, while that of a bTCP ceramic increased for several weeks and finally decreased to the level of cancellous bone via the complete degradation of the implant. On the other hand, bTCP was resorbed and remodelled more rapidly than HA in vivo. Thus, the use of bTCP might be advantageous and may result in more efficient and rapid subchondral bone remodelling after implantation.

**Figure 3:**
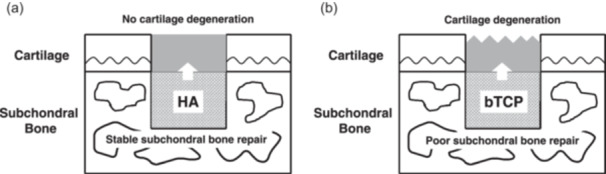
Schematic representation of the relationship between cartilage and subchondral bone repair. Hydroxyapatite (HA)‐based (a) and beta‐tricalcium phosphate (bTCP)‐based biphasic implant (b). The condition of the subchondral bone could affect the quality of overlying cartilage repair.

Notably, one recent study directly compared the material properties and healing process of ceramic bone substitutes (artificial bones), which were made of HA or bTCP, for subchondral bone repair as determined by histological and biomechanical analyses [105]. These authors have originally developed a biphasic implant composed of a mesenchymal stem cell (MSC)‐based scaffold‐free tissue‐engineered construct (TEC) as a chondral component coupled with an HA‐ or bTCP‐based artificial bone as a subchondral bone component, implanted these constructs in a rabbit osteochondral defect, and investigated its feasibility for osteochondral repair. Osteochondral defects treated with the bTCP‐based implants showed more rapid subchondral bone remodelling at an early stage after implantation, but the cartilaginous tissue deteriorated over time in a long‐term follow‐up after implantation. Similar to the histological findings, biomechanical properties also showed inferior quality with the bTCP‐based implant in a long‐term follow‐up. Osteochondral defects treated with the HA‐based implants maintained good histological quality in a long‐term follow‐up after implantation and also exhibited better biomechanical properties as compared with the bTCP‐based implants. Interestingly, the HA‐based implant facilitated better osteochondral repair than did the bTCP‐based implant. Consequently, a stable restoration of subchondral bone, rather than rapid remodelling of subchondral bone, appears fundamental for long‐term effective osteochondral repair. Also, the condition of the subchondral bone could affect the quality of the overlying cartilage repair (Figure 3), and these findings should be taken into account in the future selection of suitable materials for the treatment of patients with osteochondral lesions.

Progress in stem cell technology could potentially eliminate the need to use bone substitutes to repair osteochondral lesions. A recent study on osteochondral repair using a cartilaginous spheroid derived from human induced pluripotent stem cells (iPSCs) wrapped by the MSC–based scaffold‐free TEC demonstrated zonal osteochondral repair without the use of bone substitute [84]. Interestingly, the implantation of the iPSC‐derived cartilaginous particles itself resulted in the maintenance of cartilaginous particles within the osteochondral defect without any change since implantation. Implantation of the TEC itself resulted in the formation of fibrous tissue within the osteochondral defect. It is likely that communication between the iPSC‐cartilaginous particle and the TEC is important to proceed with zonal osteochondral repair.

## SUBCHONDRAL BONE AND OSTEOCHONDRAL SCAFFOLDS

Osteochondral scaffolds have been developed during the last 20 years, aiming at overcoming the limitations of traditional treatments for osteochondral defects. In fact, despite the good clinical outcomes reported [31], autologous osteochondral transplantation presents several limitations when addressing lesions bigger than 2.5 cm^2^ due to donor site morbidity issues [32]. On the other hand, the use of allogeneic osteochondral plugs can be considered for bigger lesions but is prone to limited availability. Even regenerative strategies, such as ACI and MACT, modified to address osteochondral defects using an autologous bone graft [30], presented a relatively high incidence of subchondral bone alterations [93], with elevated costs and morbidity related to a double surgical procedure. Osteochondral scaffolds present a bilayer structure reproducing the different biological and functional requirements of the entire osteochondral unit in order to guide the growth of both bone and cartilage tissues, respectively. The aim of such devices is to be implanted cell‐free and provide the proper stimuli to regenerate the osteochondral tissue by the in situ presence of materials that are able to support and guide cell differentiation towards bone and cartilage.

Among the many scaffolds commercialized for clinical application and specifically developed to reproduce the different biological and functional requirements of bone and cartilage, very few of them have currently been reported in the clinical literature. One of the first was a bilayer scaffold made of a porous PLGA‐calcium‐sulphate biopolymer (TruFit, Smith & Nephew) in the form of mosaic‐like cylinder plugs. After promising preclinical results, the plug was initially introduced into the clinical practice for backfilling autologous grafts donor sites, but it has been mainly studied as a direct implant for the treatment of focal articular surface defects, where it showed some controversial results [81, 117]. Dhollander et al. [27] reported a failure rate of 20% (3 out of 15 patients) at 12 months, paired with fibrous vascularized repair tissue at biopsies. The same authors questioned the use of this scaffold plugin osteochondral repair, considering the lack of evidence to support osteoconductive bone ingrowth and a failure rate of 30% [26]. Similarly, Joshi et al. reported 7 out of 10 patients being reoperated due to implant failure within the first 24 months after plug implantation for patellar lesions [48]. A comparative study with mosaicplasty, used to treat patients for similar defects, showed significantly higher outcomes for the latter ones [42]. Contrarily, a recent study on 5 patients, 64 years mean aged, reported improvement in clinical and MRI scores [15]. Nowadays, the scaffold has been withdrawn from the market.

**Figure 4:**
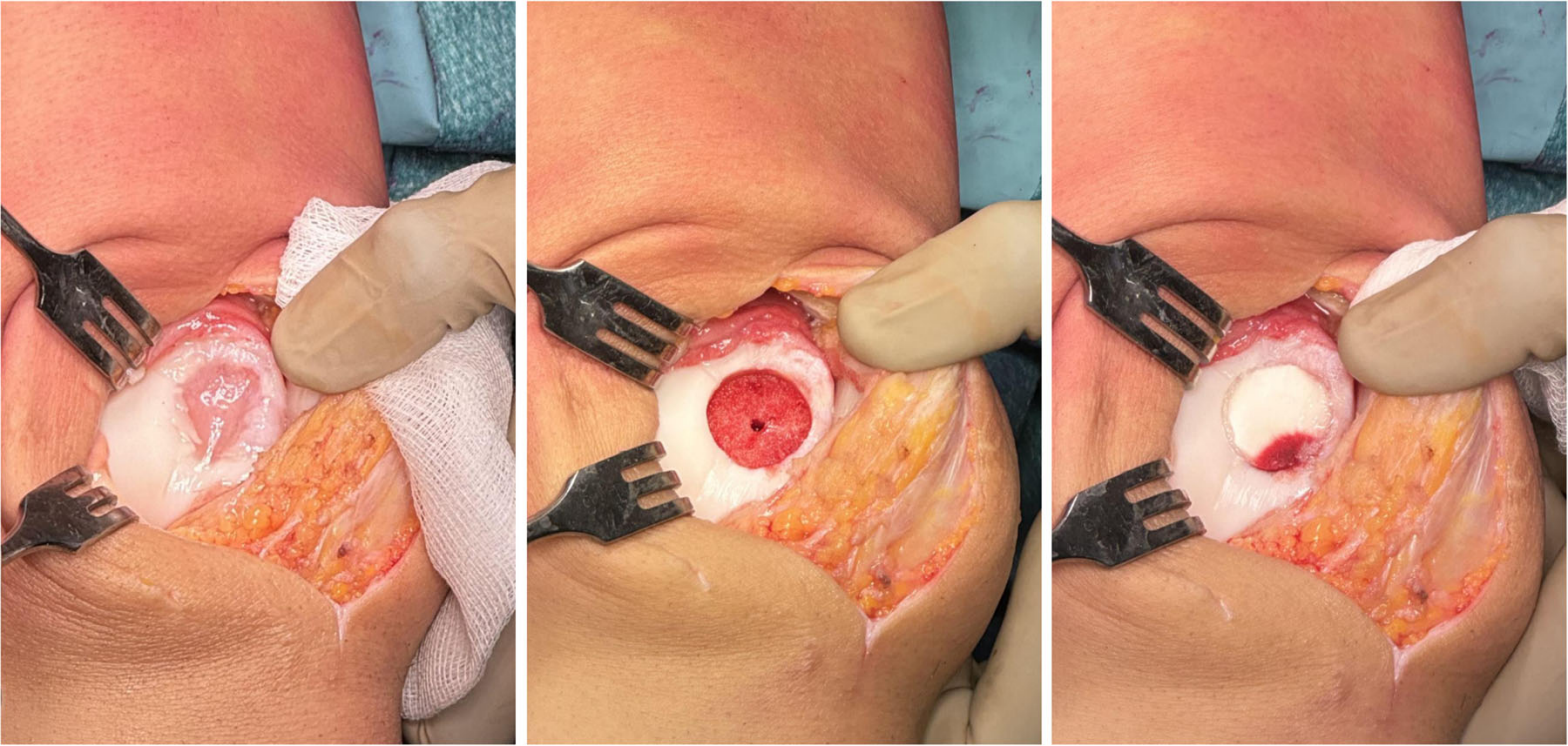
Implantation of a biomimetic osteochondral scaffold in a 22‐year‐old male patient. Left: the osteochondral defect of the femoral trochlea. Centre: the defect prepared with the dedicated instrumentation. Right: the osteochondral scaffold implanted in the defect through the press‐fit technique.

The most studied osteochondral scaffold is represented by a three‐layer nanostructured implant made of collagen and HA (MaioRegen™, FinCeramica), mimicking the composition of the extracellular matrices of cartilage and bone tissue [112] (Figure 4 shows the implantation of the scaffold with new instrumentation [4]). It showed promising results during in vitro and animal studies [56, 63], either with or without adding cells, and its clinical application has been widely reported up to mid‐term follow‐up. A study on 27 patients [60] showed significant and stable improvement in all the scores used until 60 months of follow‐up. In addition, MRI evaluation of 23 lesions revealed significant improvements in both mean MOCART score and subchondral bone status over time. Even if some abnormalities persisted, no correlation was found between imaging and clinical outcomes. Persistent subchondral bone abnormalities have been confirmed in the long term, although without a clear impact on the clinical outcome. Positive results at short‐term follow‐up were later reported also in a larger study on 79 patients [61], and the effectiveness of this approach was confirmed also in peculiar subgroups of patients, such as OCDs [29, 95], tibial plateaus [62], patella [94], large [9, 24] or complex articular lesions involving the subchondral bone [33, 98]. Eventually, this biomimetic patch was successfully applied in challenging patients affected by spontaneous osteonecrosis of the knee [10], early OA [28, 101], and as part of a combined approach to treat unicompartmental OA patients [77]. Kon et al. [59] published the results of a randomized trial comparing an osteochondral scaffold with microfractures, reporting at 2 years follow‐up similar results in chondral lesions and significantly higher scores in patients affected by osteochondral defects and in sport active patients. Besides the satisfying clinical results, concerns have been raised about the quality of regenerated tissue [12, 21, 79, 113], which may last even more than 5 years to obtain a good MRI signal [60].

More recently, an aragonite‐based osteochondral scaffold was developed and introduced in the clinical setting (Agili‐C™, CartiHeal Ltd.). It is a rigid cell‐free implant in a cylinder shape, which consists of two layers: a bone phase made of calcium carbonate in the aragonite crystalline form and a superficial cartilage phase composed of modified aragonite and hyaluronic acid. Preclinical analysis [65] showed biodegradability and intrinsic restorative potential, and the ability to recruit cells from the surrounding tissues allowed the one‐step implantation without any cell augmentation. After the first case report [58] describing the clinical use of this construct in a 47‐year‐old non‐professional sportsman resuming his pre‐injury sports activity 18 months after surgery, short‐term results of a case series of 97 patients were published [64]. At 1‐year follow‐up, patients obtained significant clinical improvement and good MRI results, with a revision rate of 8%. In the same study, the authors compared the outcomes of two different types of scaffold, cylindrical vs tapered, reporting a lower failure rate for tapered‐shaped implants. More recently, the aragonite‐based scaffold was used also in 86 patients affected by joint surface lesions in mild to moderate knee OA, with a significant clinical improvement and 9% failure rate after 24 months [11, 57]. Finally, a multicenter randomized trial versus a control group (arthroscopic debridement/microfractures) was conducted in 251 patients affected by joint surface lesions of the knee, including those with concurrent mild to moderate osteoarthritis. The aragonite‐based scaffold showed a statistically superior outcome in the primary endpoint and all secondary endpoints at each follow‐up, and the failure rate was 7.2% for the scaffold group versus 21.4% for the control [2].

Multilayered biomimetic scaffolds showed promising results in the treatment of osteochondral lesions [11], although the evidence is limited by the few number of available studies and subchondral bone regeneration still shows some limitations [54]. Future trends will include the development of new scaffolds and the improvement of the current ones, aiming at reducing the invasiveness of the surgical procedure and ameliorating the integration and regeneration of the implant, especially in the bone layer [52].

## CONCLUSIONS

Subchondral bone is gaining a central role in the understanding of articular lesions from an aetiologic point of view and as a diagnostic tool. Moreover, its status is also gaining importance as a prognostic factor in the follow‐up of chondral treatments. Finally, the recognition of its importance in the natural history of these lesions led to consider it as a treatment target, with the development of specific scaffolds and treatment procedures to address osteochondral lesions. Several aspects still need to be understood, but a growing interest in subchondral bone is to be expected in the upcoming future, and the increasing knowledge about this topic will help physicians in the diagnosis and in the treatment of alterations of the subchondral bone.

## AUTHOR CONTRIBUTIONS

**Giuseppe Filardo**: Conceptualization; writing—review and editing; supervision. **Luca Andriolo**: Conceptualization; writing—review and editing. **Elizaveta Kon**: Conceptualization; writing—review and editing. **Alessandro Sangiorgio**: Writing—original draft preparation. **Wayne Gersoff**: Writing—original draft preparation. **Norimasa Nakamura**: Writing—original draft preparation. **Stefan Nehrer**: Writing—original draft preparation. **Francesca Vannini**: Writing—original draft preparation. All authors have read and agreed to the published version of the manuscript.

## CONFLICT OF INTEREST STATEMENT

The authors declare no conflict of interest.

## ETHICS STATEMENT

No ethical committee approval or patient consent was needed due to the nature of the study.

## References

[1] Aae, T.F. , Randsborg, P.H. , Lurås, H. , Årøen, A. & Lian, Ø.B. (2018) Microfracture is more cost‐effective than autologous chondrocyte implantation: a review of level 1 and level 2 studies with 5 year follow‐up. Knee Surgery, Sports Traumatology, Arthroscopy: Official Journal of the ESSKA, 26, 1044–1052. Available from: 10.1007/s00167-017-4802-5 29128878 PMC5876257

[2] Altschuler, N. , Zaslav, K.R. , Di Matteo, B. , Sherman, S.L. , Gomoll, A.H. , Hacker, S.A. et al. (2023) Aragonite‐based scaffold versus microfracture and debridement for the treatment of knee chondral and osteochondral lesions: results of a multicenter randomized controlled trial. The American Journal of Sports Medicine, 51, 957–967. Available from: 10.1177/03635465231151252 36779614

[3] Ambra, L.F. , de Girolamo, L. & Gomoll, A.H. (2019) Pulse lavage fails to significantly reduce bone marrow content in osteochondral allografts: a histological and DNA quantification study. The American Journal of Sports Medicine, 47, 2723–2728. Available from: 10.1177/0363546519864716 31373832

[4] Andriolo, L. , De Marziani, L. , Di Martino, A. , Boffa, A. , Zaffagnini, S. & Filardo, G. (2024) Cell‐free biomimetic scaffold for chondral and osteochondral lesions: surgical technique for custom and standardized implantation. Journal of Cartilage & Joint Preservation, 4, 100173. 10.1016/j.jcjp.2024.100173

[5] Andriolo, L. , Reale, D. , Di Martino, A. , Boffa, A. , Zaffagnini, S. & Filardo, G. (2021) Cell‐free scaffolds in cartilage knee surgery: a systematic review and meta‐analysis of clinical evidence. Cartilage, 12, 277–292. Available from: 10.1177/1947603519852406 31166117 PMC8236653

[6] Apostol, L. , Boudousq, V. , Basset, O. , Odet, C. , Yot, S. , Tabary, J. et al. (2006) Relevance of 2D radiographic texture analysis for the assessment of 3D bone micro‐architecture. Medical Physics, 33, 3546–3556. Available from: 10.1118/1.2211727 17022251

[7] Baranyay, F.J. , Wang, Y. , Wluka, A.E. , English, D.R. , Giles, G.G. , Sullivan, R.O. et al. (2007) Association of bone marrow lesions with knee structures and risk factors for bone marrow lesions in the knees of clinically healthy, community‐based adults. Seminars in Arthritis and Rheumatism, 37, 112–118. Available from: 10.1016/j.semarthrit.2007.01.008 17391738

[8] Baumann, C. , Baumann, J. , Bozynski, C. , Stoker, A. , Stannard, J. & Cook, J. (2019) Comparison of techniques for preimplantation treatment of osteochondral allograft bone. The Journal of Knee Surgery, 32, 097–104. Available from: 10.1055/s-0038-1636834 29514363

[9] Berruto, M. , Delcogliano, M. , de Caro, F. , Carimati, G. , Uboldi, F. , Ferrua, P. et al. (2014) Treatment of large knee osteochondral lesions with a biomimetic scaffold: results of a multicenter study of 49 patients at 2‐year follow‐up. The American Journal of Sports Medicine, 42, 1607–1617. Available from: 10.1177/0363546514530292 24778267

[10] Berruto, M. , Ferrua, P. , Uboldi, F. , Pasqualotto, S. , Ferrara, F. , Carimati, G. et al. (2016) Can a biomimetic osteochondral scaffold be a reliable alternative to prosthetic surgery in treating late‐stage SPONK? The Knee, 23, 936–941. Available from: 10.1016/j.knee.2016.08.002 27592357

[11] Boffa, A. , Solaro, L. , Poggi, A. , Andriolo, L. , Reale, D. & Di Martino, A. (2021) Multi‐layer cell‐free scaffolds for osteochondral defects of the knee: a systematic review and meta‐analysis of clinical evidence. Journal of Experimental Orthopaedics, 8, 56. Available from: 10.1186/s40634-021-00377-4 34331140 PMC8324705

[12] Brix, M. , Kaipel, M. , Kellner, R. , Schreiner, M. , Apprich, S. , Boszotta, H. et al. (2016) Successful osteoconduction but limited cartilage tissue quality following osteochondral repair by a cell‐free multilayered nano‐composite scaffold at the knee. International Orthopaedics, 40, 625–632. Available from: 10.1007/s00264-016-3118-2 26803322

[13] Buckland‐Wright, J.C. , Lynch, J.A. & Macfarlane, D.G. (1996) Fractal signature analysis measures cancellous bone organisation in macroradiographs of patients with knee osteoarthritis. Annals of the Rheumatic Diseases, 55, 749–755. Available from: 10.1136/ard.55.10.749 8984941 PMC1010294

[14] Buckwalter, J.A. & Mankin, H.J. (1998) Articular cartilage: tissue design and chondrocyte‐matrix interactions. Instructional Course Lectures, 47, 477–486. 9571449

[15] Bugelli, G. , Ascione, F. , Dell'Osso, G. , Zampa, V. & Giannotti, S. (2018) Biphasic bioresorbable scaffold (TruFit(®)) in knee osteochondral defects: 3‐T MRI evaluation of osteointegration in patients with a 5‐year minimum follow‐up. Musculoskeletal Surgery, 102, 191–199. Available from: 10.1007/s12306-017-0522-8 29164531

[16] Burr, D.B. & Gallant, M.A. (2012) Bone remodelling in osteoarthritis. Nature Reviews Rheumatology, 8, 665–673. Available from: 10.1038/nrrheum.2012.130 22868925

[17] Case, J.M. & Scopp, J.M. (2016) Treatment of articular cartilage defects of the knee with microfracture and enhanced microfracture techniques. Sports medicine and arthroscopy review, 24, 63–68. Available from: 10.1097/JSA.0000000000000113 27135288

[18] Castañeda, S. , Roman‐Blas, J.A. , Largo, R. & Herrero‐Beaumont, G. (2012) Subchondral bone as a key target for osteoarthritis treatment. Biochemical Pharmacology, 83, 315–323. Available from: 10.1016/j.bcp.2011.09.018 21964345

[19] Chang, E.Y. , Pallante‐Kichura, A.L. , Bae, W.C. , Du, J. , Statum, S. , Wolfson, T. et al. (2014) Development of a comprehensive osteochondral allograft MRI scoring system (OCAMRISS) with histopathologic, micro‐computed tomography, and biomechanical validation. Cartilage, 5, 16–27. Available from: 10.1177/1947603513514436 24489999 PMC3904392

[20] Chen, Y. , Hu, Y. , Yu, Y.E. , Zhang, X. , Watts, T. , Zhou, B. et al. (2018) Subchondral trabecular rod loss and plate thickening in the development of osteoarthritis. Journal of Bone and Mineral Research, 33, 316–327. Available from: 10.1002/jbmr.3313 29044705

[21] Christensen, B.B. (2016) Autologous tissue transplantations for osteochondral repair. Danish Medical Journal, 63(4), B5236. 27034191

[22] Cook, J.L. (2019) Editorial commentary: bone marrow aspirate biologics for osteochondral allografts‐because we can or because we should? Arthroscopy: The Journal of Arthroscopic & Related Surgery, 35, 2445–2447. Available from: 10.1016/j.arthro.2019.04.018 31395184

[23] Cook, J.L. , Stannard, J.P. , Stoker, A.M. , Bozynski, C.C. , Kuroki, K. , Cook, C.R. et al. (2016) Importance of donor chondrocyte viability for osteochondral allografts. The American Journal of Sports Medicine, 44, 1260–1268. Available from: 10.1177/0363546516629434 26920431

[24] Delcogliano, M. , de Caro, F. , Scaravella, E. , Ziveri, G. , De Biase, C.F. , Marotta, D. et al. (2014) Use of innovative biomimetic scaffold in the treatment for large osteochondral lesions of the knee. Knee Surgery, Sports Traumatology, Arthroscopy: Official Journal of the ESSKA, 22, 1260–1269. Available from: 10.1007/s00167-013-2717-3 24146051

[25] Demange, M.K. , Minas, T. , von Keudell, A. , Sodha, S. , Bryant, T. & Gomoll, A.H. (2017) Intralesional osteophyte regrowth following autologous chondrocyte implantation after previous treatment with marrow stimulation technique. Cartilage, 8, 131–138. Available from: 10.1177/1947603516653208 28345403 PMC5358827

[26] Dhollander, A. , Verdonk, P. , Almqvist, K.F. , Verdonk, R. & Victor, J. (2015) Clinical and MRI outcome of an osteochondral scaffold plug for the treatment of cartilage lesions in the knee. Acta Orthopaedica Belgica, 81, 629–638. 26790784

[27] Dhollander, A.A.M. , Liekens, K. , Almqvist, K.F. , Verdonk, R. , Lambrecht, S. , Elewaut, D. et al. (2012) A pilot study of the use of an osteochondral scaffold plug for cartilage repair in the knee and how to deal with early clinical failures. Arthroscopy: The Journal of Arthroscopic & Related Surgery, 28, 225–233. Available from: 10.1016/j.arthro.2011.07.017 22014478

[28] Di Martino, A. , Kon, E. , Perdisa, F. , Sessa, A. , Filardo, G. , Neri, M.P. et al. (2015) Surgical treatment of early knee osteoarthritis with a cell‐free osteochondral scaffold: results at 24 months of follow‐up. Injury, 46(Suppl. 8), S33–S38. Available from: 10.1016/S0020-1383(15)30052-8 26747916

[29] Filardo, G. , Kon, E. , Di Martino, A. , Busacca, M. , Altadonna, G. & Marcacci, M. (2013) Treatment of knee osteochondritis dissecans with a cell‐free biomimetic osteochondral scaffold: clinical and imaging evaluation at 2‐year follow‐up. The American Journal of Sports Medicine, 41, 1786–1793. Available from: 10.1177/0363546513490658 23761684

[30] Filardo, G. , Kon, E. , Di Martino, A. , Perdisa, F. , Busacca, M. , Tentoni, F. et al. (2014) Is the clinical outcome after cartilage treatment affected by subchondral bone edema? Knee Surgery, Sports Traumatology, Arthroscopy, 22, 1337–1344. Available from: 10.1007/s00167-013-2813-4 24337526

[31] Filardo, G. , Kon, E. , Di Matteo, B. , Di Martino, A. & Marcacci, M. (2014) Single‐plug autologous osteochondral transplantation: results at minimum 16 years' follow‐up. Orthopedics, 37, e761–e767. Available from: 10.3928/01477447-20140825-51 25350617

[32] Filardo, G. , Kon, E. , Perdisa, F. , Balboni, F. & Marcacci, M. (2014) Autologous osteochondral transplantation for the treatment of knee lesions: results and limitations at two years' follow‐up. International Orthopaedics, 38, 1905–1912. Available from: 10.1007/s00264-014-2322-1 24663398

[33] Filardo, G. , Kon, E. , Perdisa, F. , Di Matteo, B. , Di Martino, A. , Iacono, F. et al. (2013) Osteochondral scaffold reconstruction for complex knee lesions: a comparative evaluation. The Knee, 20, 570–576. Available from: 10.1016/j.knee.2013.05.007 23810647

[34] Frank, R.M. , Cotter, E.J. , Nassar, I. & Cole, B. (2017) Failure of bone marrow stimulation techniques. Sports Medicine and arthroscopy review, 25, 2–9. Available from: 10.1097/JSA.0000000000000134 28045867

[35] Giannini, S. , Buda, R. , Pagliazzi, G. , Ruffilli, A. , Cavallo, M. , Baldassarri, M. et al. (2014) Survivorship of bipolar fresh total osteochondral ankle allograft. Foot & Ankle International, 35, 243–251. Available from: 10.1177/1071100713518503 24403348

[36] Giannini, S. , Buda, R. , Ruffilli, A. , Pagliazzi, G. , Ensini, A. , Grigolo, B. et al. (2015) Failures in bipolar fresh osteochondral allograft for the treatment of end‐stage knee osteoarthritis. Knee Surgery, Sports Traumatology, Arthroscopy, 23, 2081–2089. Available from: 10.1007/s00167-014-2961-1 24700335

[37] Giannini, S. , Buda, R. , Ruffilli, A. , Pagliazzi, G. & Vannini, F. (2013) Total femoral and tibial osteochondral allograft for remobilizing a knee after arthrodesis. Knee Surgery, Sports Traumatology, Arthroscopy, 21, 2784–2789. Available from: 10.1007/s00167-012-2214-0 23014775

[38] Giannini, S. , Mazzotti, A. & Vannini, F. (2017) Bipolar fresh total osteochondral allograft in the ankle: Is it a successful long‐term solution? Injury, 48, 1319–1324. Available from: 10.1016/j.injury.2017.05.011 28529013

[39] Gomoll, A.H. , Madry, H. , Knutsen, G. , van Dijk, N. , Seil, R. , Brittberg, M. et al. (2010) The subchondral bone in articular cartilage repair: current problems in the surgical management. Knee Surgery, Sports Traumatology, Arthroscopy, 18, 434–447. Available from: 10.1007/s00167-010-1072-x PMC2839476 20130833

[40] Gratz, K.R. , Wong, B.L. , Bae, W.C. & Sah, R.L. (2009) The effects of focal articular defects on cartilage contact mechanics. Journal of Orthopaedic Research, 27, 584–592. Available from: 10.1002/jor.20762 18979528 PMC2862585

[41] Henderson, I.J.P. & La Valette, D.P. (2005) Subchondral bone overgrowth in the presence of full‐thickness cartilage defects in the knee. The Knee, 12, 435–440. Available from: 10.1016/j.knee.2005.04.003 16153850

[42] Hindle, P. , Hendry, J.L. , Keating, J.F. & Biant, L.C. (2014) Autologous osteochondral mosaicplasty or TruFit plugs for cartilage repair. Knee Surgery, Sports Traumatology, Arthroscopy, 22, 1235–1240. Available from: 10.1007/s00167-013-2493-0 23589126

[43] Huddleston, H.P. , Wong, S.E. , Cregar, W.M. , Haunschild, E.D. , Alzein, M.M. , Cole, B.J. et al. (2021) Bone marrow lesions on preoperative magnetic resonance imaging correlate with outcomes following isolated osteochondral allograft transplantation. Arthroscopy: The Journal of Arthroscopic & Related Surgery, 37, 3487–3497. Available from: 10.1016/j.arthro.2021.04.056 33964391

[44] Huey, D.J. , Hu, J.C. & Athanasiou, K.A. (2012) Unlike bone, cartilage regeneration remains elusive. Science, 338, 917–921. Available from: 10.1126/science.1222454 23161992 PMC4327988

[45] Hunziker, E.B. (1999) Articular cartilage repair: are the intrinsic biological constraints undermining this process insuperable? Osteoarthritis and Cartilage, 7, 15–28. Available from: 10.1053/joca.1998.0159 10367012

[46] Hunziker, E.B. (2009) The elusive path to cartilage regeneration. Advanced Materials, 21, 3419–3424. Available from: 10.1002/adma.200801957 20882507 PMC2950096

[47] Jiang, C.C. , Chiang, H. , Liao, C.J. , Lin, Y.J. , Kuo, T.F. , Shieh, C.S. et al. (2007) Repair of porcine articular cartilage defect with a biphasic osteochondral composite. Journal of Orthopaedic Research, 25, 1277–1290. Available from: 10.1002/jor.20442 17576624

[48] Joshi, N. , Reverte‐Vinaixa, M. , Díaz‐Ferreiro, E.W. & Domínguez‐Oronoz, R. (2012) Synthetic resorbable scaffolds for the treatment of isolated patellofemoral cartilage defects in young patients: magnetic resonance imaging and clinical evaluation. The American Journal of Sports Medicine, 40, 1289–1295. Available from: 10.1177/0363546512441585 22491793

[49] Kamibayashi, L. , Wyss, U.P. , Cooke, T.D.V. & Zee, B. (1995) Changes in mean trabecular orientation in the medial condyle of the proximal tibia in osteoarthritis. Calcified Tissue International, 57, 69–73. Available from: 10.1007/BF00299000 7671169

[50] Kamibayashi, L. , Wyss, U.P. , Cooke, T.D.V. & Zee, B. (1995) Trabecular microstructure in the medial condyle of the proximal tibia of patients with knee osteoarthritis. Bone, 17, 27–35. Available from: 10.1016/8756-3282(95)00137-3 7577155

[51] Kellgren, J.H. & Lawrence, J.S. (1957) Radiological assessment of osteo‐arthrosis. Annals of the Rheumatic Diseases, 16, 494–502. Available from: 10.1136/ard.16.4.494 13498604 PMC1006995

[52] Kluyskens, L. , Debieux, P. , Wong, K.L. , Krych, A.J. & Saris, D.B.F. (2022) Biomaterials for meniscus and cartilage in knee surgery: state of the art. Journal of ISAKOS, 7, 67–77. Available from: 10.1136/jisakos-2020-000600 35543667

[53] Kocher, M.S. , Tucker, R. , Ganley, T.J. & Flynn, J.M. (2006) Management of osteochondritis dissecans of the knee: current concepts review. The American Journal of Sports Medicine, 34, 1181–1191. Available from: 10.1177/0363546506290127 16794036

[54] Kolar, M. & Drobnič, M. (2023) Multilayered biomimetic scaffolds for cartilage repair of the talus. A systematic review of the literature. Foot and Ankle Surgery, 29, 2–8. Available from: 10.1016/j.fas.2022.10.007 36379845

[55] Kon, E. , Delcogliano, M. , Filardo, G. , Busacca, M. , Di Martino, A. & Marcacci, M. (2011) Novel nano‐composite multilayered biomaterial for osteochondral regeneration: a pilot clinical trial. The American Journal of Sports Medicine, 39, 1180–1190. Available from: 10.1177/0363546510392711 21310939

[56] Kon, E. , Delcogliano, M. , Filardo, G. , Fini, M. , Giavaresi, G. , Francioli, S. et al. (2010) Orderly osteochondral regeneration in a sheep model using a novel nano‐composite multilayered biomaterial. Journal of Orthopaedic Research, 28, 116–124. Available from: 10.1002/jor.20958 19623663

[57] Kon, E. , Di Matteo, B. , Verdonk, P. , Drobnic, M. , Dulic, O. , Gavrilovic, G. et al. (2021) Aragonite‐based scaffold for the treatment of joint surface lesions in mild to moderate osteoarthritic knees: results of a 2‐year multicenter prospective study. The American Journal of Sports Medicine, 49, 588–598. Available from: 10.1177/0363546520981750 33481631

[58] Kon, E. , Drobnic, M. , Davidson, P.A. , Levy, A. , Zaslav, K. & Robinson, D. (2014) Chronic posttraumatic cartilage lesion of the knee treated with an acellular osteochondral‐regenerating implant: case history with rehabilitation guidelines. Journal of Sport Rehabilitation, 23, 270–275. Available from: 10.1123/JSR.2013-0054 24231791

[59] Kon, E. , Filardo, G. , Brittberg, M. , Busacca, M. , Condello, V. , Engebretsen, L. et al. (2018) A multilayer biomaterial for osteochondral regeneration shows superiority vs microfractures for the treatment of osteochondral lesions in a multicentre randomized trial at 2 years. Knee Surgery, Sports Traumatology, Arthroscopy, 26, 2704–2715. Available from: 10.1007/s00167-017-4707-3 PMC6105149 28913600

[60] Kon, E. , Filardo, G. , Di Martino, A. , Busacca, M. , Moio, A. , Perdisa, F. et al. (2014) Clinical results and MRI evolution of a nano‐composite multilayered biomaterial for osteochondral regeneration at 5 years. The American Journal of Sports Medicine, 42, 158–165. Available from: 10.1177/0363546513505434 24114751

[61] Kon, E. , Filardo, G. , Perdisa, F. , Di Martino, A. , Busacca, M. , Balboni, F. et al. (2014) A one‐step treatment for chondral and osteochondral knee defects: clinical results of a biomimetic scaffold implantation at 2 years of follow‐up. Journal of Materials Science: Materials in Medicine, 25, 2437–2444. Available from: 10.1007/s10856-014-5188-2 24599553

[62] Kon, E. , Filardo, G. , Venieri, G. , Perdisa, F. & Marcacci, M. (2014) Tibial plateau lesions. Surface reconstruction with a biomimetic osteochondral scaffold: results at 2 years of follow‐up. Injury, 45(Suppl. 6), S121–S125. Available from: 10.1016/j.injury.2014.10.035 25457331

[63] Kon, E. , Mutini, A. , Arcangeli, E. , Delcogliano, M. , Filardo, G. , Nicoli Aldini, N. et al. (2010) Novel nanostructured scaffold for osteochondral regeneration: pilot study in horses. Journal of Tissue Engineering and Regenerative Medicine, 4, 300–308. Available from: 10.1002/term.243 20049745

[64] Kon, E. , Robinson, D. , Verdonk, P. , Drobnic, M. , Patrascu, J.M. , Dulic, O. et al. (2016) A novel aragonite‐based scaffold for osteochondral regeneration: early experience on human implants and technical developments. Injury, 47(Suppl. 6), S27–S32. Available from: 10.1016/S0020-1383(16)30836-1 28040083

[65] Kon, E. , Roffi, A. , Filardo, G. , Tesei, G. & Marcacci, M. (2015) Scaffold‐based cartilage treatments: with or without cells? A systematic review of preclinical and clinical evidence. Arthroscopy: The Journal of Arthroscopic & Related Surgery, 31, 767–775. Available from: 10.1016/j.arthro.2014.11.017 25633817

[66] Kon, E. , Ronga, M. , Filardo, G. , Farr, J. , Madry, H. , Milano, G. et al. (2016) Bone marrow lesions and subchondral bone pathology of the knee. Knee Surgery, Sports Traumatology, Arthroscopy, 24, 1797–1814. Available from: 10.1007/s00167-016-4113-2 27075892

[67] Kon, E. , Vannini, F. , Buda, R. , Filardo, G. , Cavallo, M. , Ruffilli, A. et al. (2012) How to treat osteochondritis dissecans of the knee: surgical techniques and new trends: AAOS exhibit selection. Journal of Bone and Joint Surgery, 94(1–8), e1. Available from: 10.2106/JBJS.K.00748 22218387

[68] Kraus, V.B. , Feng, S. , Wang, S. , White, S. , Ainslie, M. , Brett, A. et al. (2009) Trabecular morphometry by fractal signature analysis is a novel marker of osteoarthritis progression. Arthritis & Rheumatism, 60, 3711–3722. Available from: 10.1002/art.25012 19950282 PMC3711179

[69] Leal, J. , Hones, K.M. , Hao, K.A. , Slaton, P.T. & Roach, R.P. (2024) Arthroscopy and microfracture for osteochondritis dissecans of the capitellum in adolescent athletes shows favorable return to sport: a systematic review. Arthroscopy: The Journal of Arthroscopic & Related Surgery, 40, 1325–1339. Available from: 10.1016/j.arthro.2023.08.075 37714441

[70] Levy, Y.D. , Görtz, S. , Pulido, P.A. , McCauley, J.C. & Bugbee, W.D. (2013) Do fresh osteochondral allografts successfully treat femoral condyle lesions? Clinical Orthopaedics & Related Research, 471, 231–237. Available from: 10.1007/s11999-012-2556-4 22961315 PMC3528935

[71] Li, G. , Yin, J. , Gao, J. , Cheng, T.S. , Pavlos, N.J. , Zhang, C. et al. (2013) Subchondral bone in osteoarthritis: insight into risk factors and microstructural changes. Arthritis Research & Therapy, 15, 223. Available from: 10.1186/ar4405 24321104 PMC4061721

[72] Luk, J. , Stoker, A.M. , Teixeiro, E. , Kuroki, K. , Schreiner, A.J. , Stannard, J.P. et al. (2021) Systematic review of osteochondral allograft transplant immunology: how we can further optimize outcomes. The journal of knee surgery, 34, 030–038. Available from: 10.1055/s-0040-1721670 33389738

[73] Madry, H. , Grün, U.W. & Knutsen, G. (2011) Cartilage repair and joint preservation: medical and surgical treatment options. Deutsches Arzteblatt International, 108, 669–677. Available from: 10.3238/arztebl.2011.0669 22114626 PMC3221423

[74] Madry, H. , van Dijk, C.N. & Mueller‐Gerbl, M. (2010) The basic science of the subchondral bone. Knee Surgery, Sports Traumatology, Arthroscopy, 18, 419–433. Available from: 10.1007/s00167-010-1054-z 20119671

[75] Malghem, J. , Lecouvet, F. , Vande Berg, B. , Kirchgesner, T. & Omoumi, P. (2023) Subchondral insufficiency fractures, subchondral insufficiency fractures with osteonecrosis, and other apparently spontaneous subchondral bone lesions of the knee‐pathogenesis and diagnosis at imaging. Insights into Imaging, 14, 164. Available from: 10.1186/s13244-023-01495-6 37782395 PMC10545656

[76] Marcacci, M. , Andriolo, L. , Kon, E. , Shabshin, N. & Filardo, G. (2016) Aetiology and pathogenesis of bone marrow lesions and osteonecrosis of the knee. EFORT Open Reviews, 1, 219–224. Available from: 10.1302/2058-5241.1.000044 28461951 PMC5367544

[77] Marcacci, M. , Zaffagnini, S. , Kon, E. , Marcheggiani Muccioli, G.M. , Di Martino, A. , Di Matteo, B. et al. (2013) Unicompartmental osteoarthritis: an integrated biomechanical and biological approach as alternative to metal resurfacing. Knee Surgery, Sports Traumatology, Arthroscopy, 21, 2509–2517. Available from: 10.1007/s00167-013-2388-0 23370980

[78] Marquass, B. , Somerson, J.S. , Hepp, P. , Aigner, T. , Schwan, S. , Bader, A. et al. (2010) A novel MSC‐seeded triphasic construct for the repair of osteochondral defects. Journal of Orthopaedic Research, 28, 1586–1599. Available from: 10.1002/jor.21173 20973061

[79] Mathis, D.T. , Kaelin, R. , Rasch, H. , Arnold, M.P. & Hirschmann, M.T. (2018) Good clinical results but moderate osseointegration and defect filling of a cell‐free multi‐layered nano‐composite scaffold for treatment of osteochondral lesions of the knee. Knee Surgery, Sports Traumatology, Arthroscopy: Official Journal of the ESSKA, 26, 1273–1280. Available from: 10.1007/s00167-017-4638-z 28712029

[80] McCulloch, P.C. , Kang, R.W. , Sobhy, M.H. , Hayden, J.K. & Cole, B.J. (2007) Prospective evaluation of prolonged fresh osteochondral allograft transplantation of the femoral condyle: minimum 2‐year follow‐up. The American Journal of Sports Medicine, 35, 411–420. Available from: 10.1177/0363546506295178 17261573

[81] Melton, J.T. , Wilson, A.J. , Chapman‐Sheath, P. & Cossey, A.J. (2010) TruFit CB bone plug: chondral repair, scaffold design, surgical technique and early experiences. Expert Review of Medical Devices, 7, 333–341. Available from: 10.1586/erd.10.15 20420556

[82] Minas, T. , Gomoll, A.H. , Rosenberger, R. , Royce, R.O. & Bryant, T. (2009) Increased failure rate of autologous chondrocyte implantation after previous treatment with marrow stimulation techniques. The American Journal of Sports Medicine, 37, 902–908. Available from: 10.1177/0363546508330137 19261905

[83] Murphy, R.T. , Pennock, A.T. & Bugbee, W.D. (2014) Osteochondral allograft transplantation of the knee in the pediatric and adolescent population. The American Journal of Sports Medicine, 42, 635–640. Available from: 10.1177/0363546513516747 24414553

[84] Nakagawa, S. , Ando, W. , Shimomura, K. , Hart, D.A. , Hanai, H. , Jacob, G. et al. (2023) Repair of osteochondral defects: efficacy of a tissue‐engineered hybrid implant containing both human MSC and human iPSC‐cartilaginous particles. NPJ Regenerative Medicine, 8, 59. Available from: 10.1038/s41536-023-00335-x 37857652 PMC10587071

[85] Nehrer, S. , Ljuhar, R. , Steindl, P. , Simon, R. , Maurer, D. , Ljuhar, D. et al. (2021) Automated knee osteoarthritis assessment increases physicians' agreement rate and accuracy: data from the osteoarthritis initiative. Cartilage, 13, 957S–965S. Available from: 10.1177/1947603519888793 31762295 PMC8808932

[86] Neri, S. , Vannini, F. , Desando, G. , Grigolo, B. , Ruffilli, A. , Buda, R. et al. (2013) Ankle bipolar fresh osteochondral allograft survivorship and integration: transplanted tissue genetic typing and phenotypic characteristics. The Journal of Bone & Joint Surgery, 95, 1852–1860. Available from: 10.2106/JBJS.L.01715 24132359

[87] Niethammer, T.R. , Valentin, S. , Gülecyüz, M.F. , Roßbach, B.P. , Ficklscherer, A. , Pietschmann, M.F. et al. (2015) Bone marrow edema in the knee and its influence on clinical outcome after matrix‐based autologous chondrocyte implantation: results after 3‐year follow‐up. The American Journal of Sports Medicine, 43, 1172–1179. Available from: 10.1177/0363546515573935 25784628

[88] Nordström, D.C. , Santavirta, S. , Aho, A. , Heikkilä, J. , Teppo, A.M. & Konttinen, Y.T. (1999) Immune responses to osteoarticular allografts of the knee—cytokine studies. Archives of Orthopaedic and Trauma Surgery, 119, 195–198. Available from: 10.1007/s004020050389 10392518

[89] Oladeji, L.O. , Stannard, J.P. , Cook, C.R. , Kfuri, M. , Crist, B.D. , Smith, M.J. et al. (2017) Effects of autogenous bone marrow aspirate concentrate on radiographic integration of femoral condylar osteochondral allografts. The American Journal of Sports Medicine, 45, 2797–2803. Available from: 10.1177/0363546517715725 28737949

[90] Orth, P. , Cucchiarini, M. , Kohn, D. & Madry, H. (2013) Alterations of the subchondral bone in osteochondral repair‐‐translational data and clinical evidence. European Cells and Materials, 25, 299–316. Available from: 10.22203/eCM.v025a21 23813020

[91] Orth, P. , Goebel, L. , Wolfram, U. , Ong, M.F. , Gräber, S. , Kohn, D. et al. (2012) Effect of subchondral drilling on the microarchitecture of subchondral bone: analysis in a large animal model at 6 months. The American Journal of Sports Medicine, 40, 828–836. Available from: 10.1177/0363546511430376 22223716

[92] Orth, P. , Peifer, C. , Goebel, L. , Cucchiarini, M. & Madry, H. (2015) Comprehensive analysis of translational osteochondral repair: focus on the histological assessment. Progress in Histochemistry and Cytochemistry, 50, 19–36. Available from: 10.1016/j.proghi.2015.10.001 26515165

[93] Pape, D. , Filardo, G. , Kon, E. , van Dijk, C.N. & Madry, H. (2010) Disease‐specific clinical problems associated with the subchondral bone. Knee Surgery, Sports Traumatology, Arthroscopy, 18, 448–462. Available from: 10.1007/s00167-010-1052-1 20151111

[94] Perdisa, F. , Filardo, G. , Sessa, A. , Busacca, M. , Zaffagnini, S. , Marcacci, M. et al. (2017) One‐step treatment for patellar cartilage defects with a cell‐free osteochondral scaffold: a prospective clinical and MRI evaluation. The American Journal of Sports Medicine, 45, 1581–1588. Available from: 10.1177/0363546517694159 28263667

[95] Perdisa, F. , Kon, E. , Sessa, A. , Andriolo, L. , Busacca, M. , Marcacci, M. et al. (2018) Treatment of knee osteochondritis dissecans with a cell‐free biomimetic osteochondral scaffold: clinical and imaging findings at midterm follow‐up. The American Journal of Sports Medicine, 46, 314–321. Available from: 10.1177/0363546517737763 29100468

[96] Podsiadlo, P. , Dahl, L. , Englund, M. , Lohmander, L.S. & Stachowiak, G.W. (2008) Differences in trabecular bone texture between knees with and without radiographic osteoarthritis detected by fractal methods. Osteoarthritis and Cartilage, 16, 323–329. Available from: 10.1016/j.joca.2007.07.010 17825585

[97] Pritzker, K.P.H. , Gay, S. , Jimenez, S.A. , Ostergaard, K. , Pelletier, J.P. , Revell, P.A. et al. (2006) Osteoarthritis cartilage histopathology: grading and staging. Osteoarthritis and Cartilage, 14, 13–29. Available from: 10.1016/j.joca.2005.07.014 16242352

[98] Ricci, M. , Tradati, D. , Maione, A. , Uboldi, F.M. , Usellini, E. & Berruto, M. (2021) Cell‐free osteochondral scaffolds provide a substantial clinical benefit in the treatment of osteochondral defects at a minimum follow‐up of 5 years. Journal of Experimental Orthopaedics, 8, 62. Available from: 10.1186/s40634-021-00381-8 34398364 PMC8368912

[99] Roemer, F.W. , Frobell, R. , Hunter, D.J. , Crema, M.D. , Fischer, W. , Bohndorf, K. et al. (2009) MRI‐detected subchondral bone marrow signal alterations of the knee joint: terminology, imaging appearance, relevance and radiological differential diagnosis. Osteoarthritis and Cartilage, 17, 1115–1131. Available from: 10.1016/j.joca.2009.03.012 19358902

[100] Seow, D. , Yasui, Y. , Hutchinson, I.D. , Hurley, E.T. , Shimozono, Y. & Kennedy, J.G. (2019) The subchondral bone is affected by bone marrow stimulation: a systematic review of preclinical animal studies. Cartilage, 10, 70–81. Available from: 10.1177/1947603517711220 28573889 PMC6376565

[101] Sessa, A. , Andriolo, L. , Di Martino, A. , Romandini, I. , De Filippis, R. , Zaffagnini, S. et al. (2019) Cell‐free osteochondral scaffold for the treatment of focal articular cartilage defects in early knee OA: 5 years' follow‐up results. Journal of Clinical Medicine, 8, 1978. Available from: 10.3390/jcm8111978 31739539 PMC6912384

[102] Shea, K.G. , Jacobs, Jr., J.C. , Carey, J.L. , Anderson, A.F. & Oxford, J.T. (2013) Osteochondritis dissecans knee histology studies have variable findings and theories of etiology. Clinical Orthopaedics & Related Research, 471, 1127–1136. Available from: 10.1007/s11999-012-2619-6 23054514 PMC3586021

[103] Shimomura, K. , Moriguchi, Y. , Ando, W. , Nansai, R. , Fujie, H. , Hart, D.A. et al. (2014) Osteochondral repair using a scaffold‐free tissue‐engineered construct derived from synovial mesenchymal stem cells and a hydroxyapatite‐based artificial bone. Tissue Engineering. Part A, 20, 2291–2304. Available from: 10.1089/ten.tea.2013.0414 24655056

[104] Shimomura, K. , Moriguchi, Y. , Murawski, C.D. , Yoshikawa, H. & Nakamura, N. (2014) Osteochondral tissue engineering with biphasic scaffold: current strategies and techniques. Tissue Engineering Part B: Reviews, 20, 468–476. Available from: 10.1089/ten.teb.2013.0543 24417741

[105] Shimomura, K. , Moriguchi, Y. , Nansai, R. , Fujie, H. , Ando, W. , Horibe, S. et al. (2017) Comparison of 2 different formulations of artificial bone for a hybrid implant with a tissue‐engineered construct derived from synovial mesenchymal stem cells: a study using a rabbit osteochondral defect model. The American Journal of Sports Medicine, 45, 666–675. Available from: 10.1177/0363546516668835 28272938

[106] Shimozono, Y. , Hurley, E.T. , Yasui, Y. , Deyer, T.W. & Kennedy, J.G. (2018) The presence and degree of bone marrow edema influence midterm clinical outcomes after microfracture for osteochondral lesions of the talus. The American Journal of Sports Medicine, 46, 2503–2508. Available from: 10.1177/0363546518782701 30015509

[107] Sledge, S.L. (2001) Microfracture techniques in the treatment of osteochondral injuries. Clinics in Sports Medicine, 20, 365–378. Available from: 10.1016/S0278-5919(05)70311-2 11398363

[108] Stannard, J.P. & Cook, J.L. (2020) Prospective assessment of outcomes after primary unipolar, multisurface, and bipolar osteochondral allograft transplantations in the knee: a comparison of 2 preservation methods. The American Journal of Sports Medicine, 48, 1356–1364. Available from: 10.1177/0363546520907101 32134685

[109] Stoker, A. , Baumann, C. , Stannard, J. & Cook, J. (2018) Bone marrow aspirate concentrate versus platelet rich plasma to enhance osseous integration potential for osteochondral allografts. The journal of knee surgery, 31, 314–320. Available from: 10.1055/s-0037-1603800 28646824

[110] Stoker, A.M. , Stannard, J.P. , Kuroki, K. , Bozynski, C.C. , Pfeiffer, F.M. & Cook, J.L. (2018) Validation of the missouri osteochondral allograft preservation system for the maintenance of osteochondral allograft quality during prolonged storage. The American Journal of Sports Medicine, 46, 58–65. Available from: 10.1177/0363546517727516 28937783

[111] Takahashi, T. , Tins, B. , McCall, I.W. , Richardson, J.B. , Takagi, K. & Ashton, K. (2006) MR appearance of autologous chondrocyte implantation in the knee: correlation with the knee features and clinical outcome. Skeletal Radiology, 35, 16–26. Available from: 10.1007/s00256-005-0002-3 16284767

[112] Tampieri, A. , Sandri, M. , Landi, E. , Pressato, D. , Francioli, S. , Quarto, R. et al. (2008) Design of graded biomimetic osteochondral composite scaffolds. Biomaterials, 29, 3539–3546. Available from: 10.1016/j.biomaterials.2008.05.008 18538387

[113] Verdonk, R. , Goubau, Y. , Almqvist, F.K. & Verdonk, P. (2015) Biological methods to enhance bone healing and fracture repair. Arthroscopy: The Journal of Arthroscopic & Related Surgery, 31, 715–718. Available from: 10.1016/j.arthro.2014.11.045 25682328

[114] Walsh, D.A. , Sofat, N. , Guermazi, A. & Hunter, D.J. (2023) Osteoarthritis bone marrow lesions. Osteoarthritis and Cartilage, 31, 11–17. Available from: 10.1016/j.joca.2022.09.007 36191832

[115] Wang, D. , Lin, K.M. , Burge, A.J. , Balazs, G.C. & Williams 3rd, R.J. (2019) Bone marrow aspirate concentrate does not improve osseous integration of osteochondral allografts for the treatment of chondral defects in the knee at 6 and 12 months: a comparative magnetic resonance imaging analysis. The American Journal of Sports Medicine, 47, 339–346. Available from: 10.1177/0363546518813915 30543757

[116] Wang, X. , Ren, Z. , Liu, Y. , Ma, Y. , Huang, L. , Song, W. et al. (2023) Characteristics and clinical outcomes after osteochondral allograft transplantation for treating articular cartilage defects: systematic review and single‐arm meta‐analysis of studies from 2001 to 2020. Orthopaedic Journal of Sports Medicine, 11, 23259671231199418. Available from: 10.1177/23259671231199418 37745815 PMC10515554

[117] Williams, R.J. & Gamradt, S.C. (2008) Articular cartilage repair using a resorbable matrix scaffold. Instructional Course Lectures, 57, 563–571. 18399610

[118] Yamasaki, N. , Hirao, M. , Nanno, K. , Sugiyasu, K. , Tamai, N. , Hashimoto, N. et al. (2009) A comparative assessment of synthetic ceramic bone substitutes with different composition and microstructure in rabbit femoral condyle model. Journal of Biomedical Materials Research, Part B: Applied Biomaterials, 91, 788–798. Available from: 10.1002/jbm.b.31457 19572298

